# EARLY OUTCOMES OF A NATIONAL CONSORTIUM TO ADVANCE FAMILY CAREGIVERS IN NURSING EDUCATION

**DOI:** 10.1093/geroni/igac059.2756

**Published:** 2022-12-20

**Authors:** Hui Zhao, Lindsay Mullins, Andra Davis, Kathryn Sexson, Sara Hart

**Affiliations:** James Madison University, Harrisonburg, Virginia, United States; Franciscan Missionaries of Our Lady University, Baton Rouge, Louisiana, United States; University of Portland, Portland, Oregon, United States; UC Davis, Sacramento, California, United States; University of Utah, Salt Lake City, Utah, United States

## Abstract

Family caregivers (FCGs) hold primary responsibility in caring for people with chronic illness often with little training and insufficient resources Standardized guidance for supporting FCGs currently does not exist. Given this fact, it is an urgent call to healthcare professionals, particularly nurses as the key members of the healthcare team, to promote education for FCGs. In April 2022, experts in the field of family caregiving from UC Davis and University of Utah schools of nursing, convened educators and champions of FCGs from across the country and formed the Family Caregiving Nursing Education Consortium to advance the inclusion of FCGs in nursing education in order to address the lack of standardized nursing competencies for family caregiving. The consortium consists of members from nine diverse universities representing member expertise in pediatrics, geriatrics, palliative care, disabilities, mental health, and underserved communities in order to address a broader range of health issues through the lifespan. Preliminary data were reviewed and identified as a framework, including Family Caregiver Competencies for Interprofessional Education (4 domains) developed by the school of nursing at UC Davis and the AACN Essentials (10 competency categories). Members mapped the family caregiving competencies to the AACN Essentials (2021) during monthly meetings. Though current work presented an early-stage outcome, it marched through crucial steps, including institutional environmental scans, needs assessments, and toolkit development, as building blocks that not only advanced the inclusion of family caregiving education in nursing curricula but also provided foundation to guide the next stage of work.

